# The NIST 30 MHz Linear Measurement System

**DOI:** 10.6028/jres.099.003

**Published:** 1994

**Authors:** Jeffrey A. Jargon, Ronald A. Ginley, Douglas D. Sutton

**Affiliations:** National Institute of Standards and Technology, Boulder, CO 80303-3328

**Keywords:** attenuation, automated, calibration, linear, measurement, power, thermistor, uncertainty

## Abstract

An automated linear measurement system (LMS) has been developed to determine the nonlinearity of a tuned 30 MHz power detector over a 6.021 dB range. This detector uses a single thermistor bead design with thermal isolation to obtain nearly linear tracking over a 4:1 change in input power. The nonlinear correction for this change, determined by the LMS, is on the order of 1.00030 (+130 μB) for the detector presently in use. Initial experiments indicate an expanded uncertainty of ±0.138% (±598 μB), which is based upon Type A and Type B components.

## 1. Introduction

There has been a recent interest in and demand for a calibration service at NIST to support rf attenuators and voltage doublers that operate specifically at 30 MHz, The first step required to offer such a service is to develop a reference standard. For the best possible accuracy, a tuned single-element thermistor mount was chosen. A linear measurement system was designed and constructed at NIST to calibrate the nonlinearity of this mount. This paper contains a description of the LMS, an explanation of the measurement scheme, calibration results, and an uncertainty analysis.

## 2. System Description

**Fig. 1:**
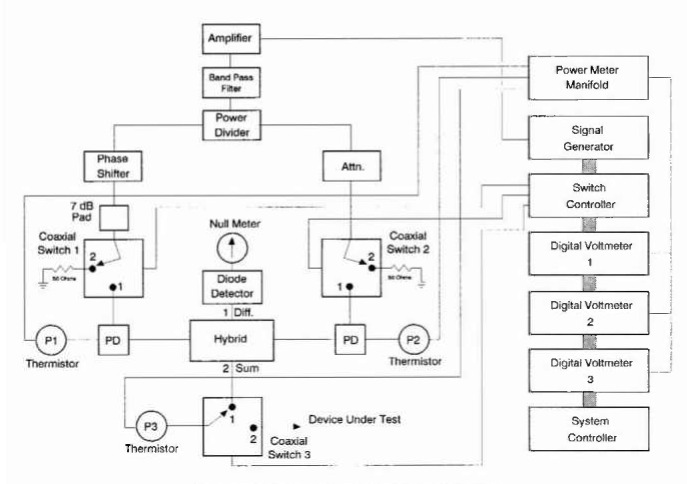
Block diagram of 30 MHz linear measurement system.

A diagram of the system is shown in Fig. 1. The signal generator provides a stable 30 MHz rf signal that is amplified and Filtered before the signal is split into two channels. One channel consists of a variable phase shifter and a fixed attenuator. A coaxial switch either terminates the signal with a 50 Ω termination or feeds the signal into a power divider, which splits the signal again. Half of the signal is detected by a single-element thermistor mount, P_1_, and the other half is fed into the hybrid. The second channel consists of a variable attenuator. Like the first channel, a coaxial switch either terminates the signal with a 50 Ω load or feeds the signal into a power divider, which splits the signal. Half of the signal is detected by a single-element thermistor mount, P_2_, and the other half is fed into the hybrid.

The hybrid takes the sum and difference of the two input signals. The difference is fed into a diode detector to rectify the signal, and then into a null meter. The sum is fed into a coaxial switch, and when the switch is in position 1, the signal is detected by a third thermistor mount, P_3_, which is the thermistor to be calibrated.

Each thermistor mount is connected to a NIST Type IV bridge and a digital voltmeter to measure rf power.

The computer controls the signal generator, the digital voltmeters, and the switch controller, and handles the data acquisition and processing through an IEEE-488 bus.

## 3. Design of 30 MHz Single-Element Thermistor Mount

The dc response of detector P_3_ must be nearly linear with changes in input rf power. A single 50 Ω thermistor bead design was selected as a linear detection scheme [1]. This detector is used in conjunction with a NIST Type IV self-balancing dc-substitution rf power meter modified to bias a 50 Ω detector [2]. The Type IV power meter is designed to change the thermistor bead bias current so that the thermistor always maintains the same resistance. The detectors are placed in the LMS housing where the temperature is held to ±0.2 °C.

It is difficult to filter the rf signal from the power meter leads due to the nature of the single bead. The total rf signal should appear across the bead in the ideal case. Other considerations in the detector design include thermal stability and any forms of rf leakage into or out of the detector.

**Fig. 2:**
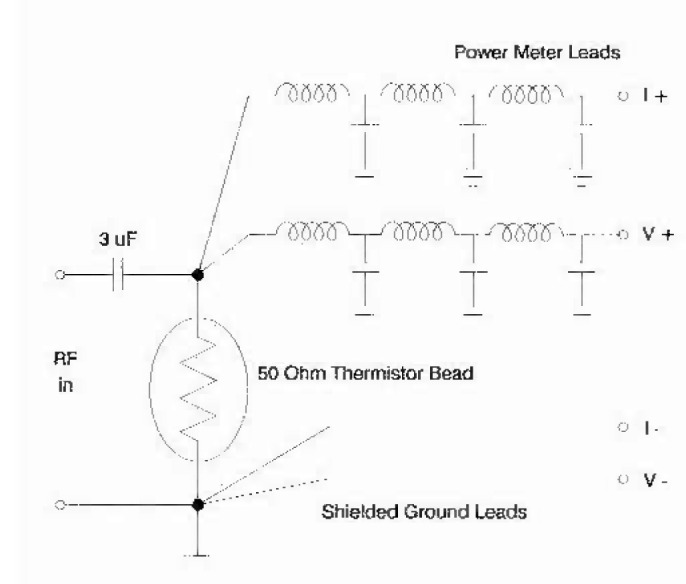
Circuit diagram of single-clement thermistor mount. All inductors are hand-wound and all capacitors are 1 μF unless otherwise labeled.

Several ideas are incorporated in the detector to eliminate these problems. Figure 2 shows the circuit diagram of a single-element thermistor mount. LC filter sections are inserted to filter the rf signal in the power meter leads. No ferrite material (such as a ferrite core inductor) is used near the thermistor, since ferrite components experience changes in impedance with changes in the rf power. These impedance changes lead to nonlinearities in the detector response. Therefore, air-cored inductors are inserted near the thermistor. Ferrite-cored conductors are allowable in sections following the first filter section because the rf power is sufficiently reduced and renders any impedance variation negligible. The parallel LC filter is tuned to resonate at 30 MHz. A special thermistor-mounting structure has been developed, and consists of an electrically insulated doughnut and two copper blocks, one on either side of the doughnut. The thermistor is placed inside the doughnut hole and is encapsulated in an air pocket by the addition of the copper blocks. The copper blocks also provide a large thermal mass so that the entire structure cannot experience rapid changes in temperature. The thermal time constant is much longer than the time required to perform a single measurement cycle. Double-sided copper-clad fiberglass boards are used in the exterior detector housing and in the internal compartment walls which separate various filter sections. This type of construction and the use of capacitive feedthroughs in the compartment walls reduce the amount of rf leakage though the detector. These physical and electrical construction considerations provide a stable linear detector with low rf leakage.

## 4. Measurement Methods

### 4.1. Calibration of P_3_

Three power meters are used in the LMS–P_1_, P_2_, and P_3_. When a given power meter is read, the notation used is *P*_XYT_, where *X* denotes the power meter (1, 2, or 3), *Y* is 1 if channel 1 is switched on and 0 if channel 1 is switched off, *Z* is 1 if channel 2 is switched on and 0 if channel 2 is switched off, and “r” denotes that this power is a reading and not a true value. Powers without a subscript “r” are true values.

The calibration of P_3_ is achieved using a three-stage method. First, with switches 1, 2, and 3 each in position 1, enough power is applied so that a nominal 12 mW is incident on the three thermistor mounts. The phase shifter and variable attenuator are adjusted to balance the two channels, thus obtaining a null on the null meter. Readings are taken on P_1_, P_2_, and P_3_ and are designated *P*_111r_, *P*_211r_, and P_311r_, respectively. Next, switch 2 is moved to position 2, so that power is only applied to the first channel. Readings are taken on P_1_ and F_3_ and are designated *P*_110r_ and *P*_310r_, respectively, A nominal 12 mW will be incident on P_1_ and approximately 3 mW will be incident on P_3_. Finally, switch 2 is moved back to position 1 and switch 1 is moved to position 2, so that power is only applied to the second channel. Readings are taken on P_2_ and P_3_ and are designated *P*_201r_ and *P*_310r_, respectively. A nominal 12 mW will be incident on P_2_ and approximately 3 mW will be incident on P_3_.

The calibration constant of P_3_, denoted $C_{\mathrm{HL}}^{2}$, is calculated using

$$C_{\mathrm{HL}}^{2}={\left[{\left(\frac{\frac{P_{310r}}{P_{110r}}}{\frac{P_{311r}}{P_{111r}}}\right)}^{1/2}+{\left(\frac{\frac{P_{301r}}{P_{201r}}}{\frac{P_{311r}}{P_{211r}}}\right)}^{1/2}\right]}^{2}.$$ (1)

The derivation of this formula can be found in Appendix A. The calibration constant is a measure of the detector’s nonlinearity over the 6.021 dB power change, and is used as a multiplication factor to correct the ratio measured by P_3_, where

$$\frac{P_{311}}{P_{310}}=C_{\mathrm{HL}}^{2}\frac{{P_{311}}_{r}}{P_{310r}}.$$ (2)

### 4.2. Power Measurements

**Fig. 3:**
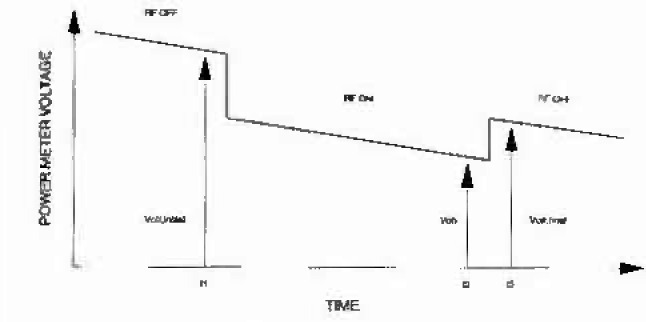
Sequence for measuring power meter dc voltages.

The NIST Type IV power meter must be connected to an external dc voltmeter. The substituted dc power, *P*_dc_, is calculated from measured voltages using

$$P_{\mathrm{dc}}=\frac{V_{\text{off}}^{2}-V_{\text{on}}^{2}}{R_{0}},$$ (3)

where *V*_off_ is the output voltage with no rf power applied, *V*_on_ is the output voltage with rf applied, and *R*_0_ (50 Ω) is the resistance of the thermistor mount. Figure 3 shows the measurement sequence for a power calculation [3]. An initial *V*_off_ is taken; rf power is applied and *V*_on_ is measured; rf power is removed and a final *V*_off_ is taken. The initial and final dc measurements are used with the *V*_on_ measurement to calculate the power and correct for any mount drift, which is assumed to be linear. The calculated value of *V*_off_ in Eq. (3) is given by

$$V_{\text{off}}=V_{\text{off},i}+\frac{t_{2}-t_{1}}{t_{3}-t_{1}}\left(V_{\text{off},f}-V_{\text{off},i}\right),$$ (4)

where *V*_off,i_ is the voltage reading taken before rf is applied at time *t*_1_ *V*_off,f_ is the voltage taken after rf is removed at time *t*_3_, and *t*_2_ is the time at which *V*_on_ is taken.

## 5. Results

The calibration constant, $C_{\mathrm{HL}}^{2}$, which is a measure of the nonlinearity of mount P_3_, was obtained by repeated measurements. The average value of the three hundred trials taken so far is $C_{\mathrm{HL}}^{2}=1.00030$ or +130 μB. Long-term data are being accumulated to validate the calibration of P_3_.

Table 1 shows a sample calibration. Powers read by the three thermistor mounts are displayed for each of the three stages. The calculated calibration constant is shown, along with the actual step in power for each leg of the system.

## 6. Uncertainty Analysis

### 6.1. Evaluation of Type A Standard Uncertainty

Evaluation of a Type A standard uncertainty may be based on any valid statistical method for treating data. Examples are calculating the standard deviation of the mean of a series of independent observations, using the method of least squares to fit a curve to data in order to estimate the parameters of the curve and their standard deviations, and carrying out an analysis of variance in order to identify and quantify random effects in certain kinds of measurements [4].

The calibration of the standard mount, P_3_, has been repeated three hundred times to determine the repeatability of the system. Tests were performed at various times of the day over several days to cover as many random factors as possible, including variations of environmental conditions and the operator’s ability to renull the system. The sample standard deviation of the mean is ±0.000335% or ±2 μB. Long-term data are being accumulated to validate this calibration, and a control chart is being developed to track any possible outliers or drift.

### 6.2. Evaluation of Type B Standard Uncertainty

Evaluation of a Type B standard uncertainty is based upon scientific judgment using all of the available relevant information. This includes previous measurement data, manufacturers’ specifications, data provided in calibration reports, knowledge of the behavior of relevant instruments and materials, and uncertainties assigned to reference data taken from handbooks [4].

The Type B evaluation of standard uncertainty accounts for the following factors:

- Uncertainty in the dc voltmeter measurements.
- Uncertainty in the Type IV power meters.
- Imperfect isolation between the two legs.
- Uncertainty because *P*_301_*≠P*_310_
- Effects of impedance changes in P_3_.
- RF leakage.
- Spurious signals and harmonics.

#### 6.2.1 Voltmeter Uncertainty

The uncertainty in the individual voltmeter readings may be determined by taking the total differential of the power expression, Eq. (3), which gives

$$dP=\frac{2}{R_{0}}\left(V_{\text{off}}dV_{\text{off}}-V_{\text{on}}dV_{\text{on}}\right).$$ (5)

The total differential of power, Eq. (5), may be determined by taking the differential of *V*_off_, Eq. (4), which gives

$$dV_{\text{off}}=\left(1-T\right)dV_{\text{off},i}+TdV_{\text{off},f},$$ (6)

where

$$T=\frac{t_{2}-t_{1}}{t_{3}-t_{1}}.$$ (7)

The uncertainties, d*V*_off,i_. d*V*_off,f_, and d*V*_on_, in the measured values of *V*_off,i_, *V*_off,r_, and *V*_on_, are based on the voltmeter manufacturer’s specifications.

**Fig. 4:**
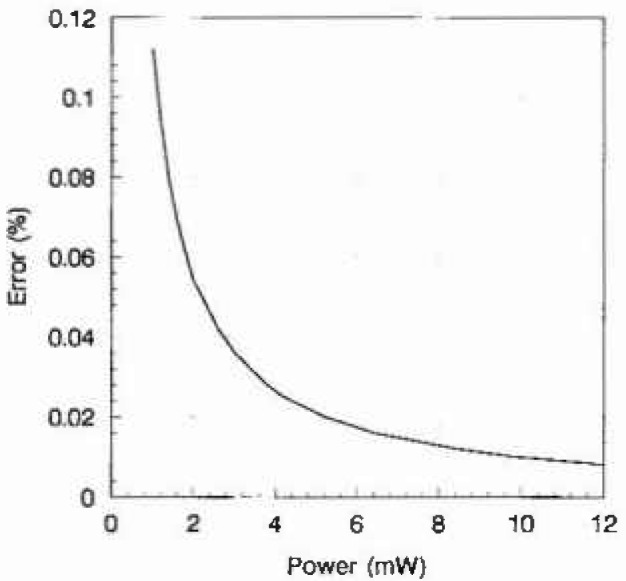
Power measurement uncertainty duc to DVM when ratios are taken.

Figure 4 shows the uncertainty in the power measurement as a function of power level, assuming the powers are ratioed as they are in the $C_{\mathrm{HL}}^{2}$ calculation, Eq. (1). The power measurements, *P*_301_ and *P*_310_, which are approximately 3 mW, result in uncertainties of 0.036%. The other power measurements, which are approximately 12 mW, result in uncertainties of 0.008%. The uncertainty of $C_{\mathrm{HL}}^{2}$ due to the voltmeter readings may be found by inserting the individual power uncertainties into Eq. (1), which gives

$$C_{\mathrm{HL}}^{2}+\Delta_{v}={\left[{\left(\frac{\frac{P_{310}+\Delta_{310}}{P_{110}-\Delta_{110}}}{\frac{P_{311}+\Delta_{311}}{P_{111}-\Delta_{111}}}\right)}^{1/2}+{\left(\frac{\frac{P_{301}+\Delta_{301}}{P_{201}-\Delta_{201}}}{\frac{P_{311}+\Delta_{311}}{P_{211}-\Delta_{211}}}\right)}^{1/2}\right]}^{2}.$$ (8)

This results in an uncertainly of Δ*r* = ±0.028% or ± 122 μB.

### 6.2.2 Type IV Powcr-Mctcr Uncertainty

The four possible sources of uncertainties internal to the Type IV power meter are the reference resistors, the operational amplifier open-loop gain, input offset voltage, and input bias current. Larsen has shown that the uncertainties due to the Type IV power meters are negligible compared to those of the voltmeters [2].

#### 6.2.3 Imperfect Isolation

An uncertainty in the calculation of $C_{\mathrm{HL}}^{2}$ may result if P_1_ and P_2_ are not zero when they are assumed to be. The thermistor mounts are square-law detectors and are not sufficiently sensitive to determine whether P_1_ and P_2_ are low enough in the “off” condition to avoid significant errors. This uncertainty is derived in Appendix B.

The corrected formula for $C_{\mathrm{HL}}^{2}$, taking imperfect isolation into account, is

$$C_{\mathrm{HL}}^{2}={\left[{\left(\frac{\frac{P_{310r}}{P_{110r}}}{\frac{P_{311r}}{P_{111r}}}\right)}^{1/2}\frac{1}{\left|1+\beta_{1}\right|}+{\left(\frac{\frac{P_{301r}}{P_{201r}}}{\frac{P_{311r}}{P_{211r}}}\right)}^{1/2}\frac{1}{\left|1+\gamma \right|}\right]}^{2},$$ (9)

where

$$\beta_{1}=-\frac{Q_{12}\Delta b_{2}}{b_{110}}$$ (10)

and

$$\gamma_{1}=-\frac{\Delta b_{1}}{b_{201}Q_{12}}.$$ (11)

Here, γ_1_ is the measure of P_1_’s contribution to $C_{\mathrm{HL}}^{2}$ if it is not zero when it is assumed to be, and *β*_1_ is the measure of P_2_’s contribution to $C_{\mathrm{HL}}^{2}$ if it is not zero when it is assumed to be. The terms, *b*_110_ and *b*_201_ are the corresponding voltages to the powers *P*_110_ and *P*_201_, respectively. Assuming *P*_110_ and *P*_201_ are 12 mW, *b*_110_ and *b*_201_ are equal to 0.7746 V. The term, *Q*_12_, is defined in Appendix B and its value is approximately −1. The Δ*b*’s represent the *b*’s which were assumed to be zero in Appendix A. If the isolation between the channels is 65 dB, as is stated in the power divider manufacturer’s specifications, then Δ*b*_1_ = Δ*b*_2_ = 0.000436. Using nominal values for the *P*’s and substituting the values into Eq. (9), an uncertainty of ± 0.112% or ± 488 μB is obtained.

#### 6.2.4 Uncertainty Because *P*_301_*≠P*_310_

An uncertainty in the calculation of $C_{\mathrm{HL}}^{2}$ may result if *P*_301_*≠P*_310._ The derivation of this uncertainty can be found in Appendix C.

The measure of nonlinearity, *α*, of thermistor mount three is

$$\alpha =\frac{1-C_{\mathrm{HL}}^{2}}{C_{\mathrm{HL}}^{2}P_{\mathrm{Lr}}-P_{\mathrm{Hr}}},$$ (12)

where *P*_Lr_ is the reading of mount three at the low level and *P*_Hr_, is the power reading of mount three at the high level. Assuming $C_{\mathrm{HL}}^{2}=1.0004$ worst case, *P*_Lr_ =3 mW, and P_Hr_ = 12 mW, *α* is calculated to be 3.334 × 10^−5^. The ratio *K*_301_/*K*_310_ is given by

$$\frac{K_{301}}{K_{310}}=\frac{1+\alpha P_{301r}}{1+\alpha P_{310r}}.$$ (13)

If *P*_301r_ is 10% greater than *P*_310r_, then

$$\frac{K_{301}}{K_{310}}=\frac{1+\alpha \left(3\times 1.10\right)}{1+\alpha \left(3\right)}=1.000010.$$ (14)

The effect of this being nonunity results in a corrected formula for $C_{\mathrm{HL}}^{2}$, where

$$C_{\mathrm{HL}}^{2}={\left[{\left(\frac{\frac{P_{310r}}{P_{110r}}}{\frac{P_{311r}}{P_{111r}}}\right)}^{1/2}+{\left(\frac{\frac{K_{301}}{K_{310}}\frac{P_{310r}}{P_{201r}}}{\frac{P_{311r}}{P_{211r}}}\right)}^{1/2}\right]}^{2}=1.000005$$ (15)

using nominal values for the P’s. The result is an uncertainty of ±0.0005% or ±3 μB.

#### 6.2.5 Uncertainty Due to Impedance Changes in *P*_3_

An uncertainty in the calculation of $C_{\mathrm{HL}}^{2}$. may result if the impedance changes in P_3_ with power level. The derivation of this uncertainty can be found in Appendix D.

The corrected formula for $C_{\mathrm{HL}}^{2}$, taking impedance changes in P_3_ into account, is

$$C_{\mathrm{HL}}^{2}=\frac{{\left[{\left(\frac{\frac{P_{310r}}{P_{110r}}}{\frac{P_{311r}}{P_{111r}}}\right)}^{1/2}+{\left(\frac{\frac{P_{310r}}{P_{201r}}}{\frac{P_{311r}}{P_{211r}}}\right)}^{1/2}\right]}^{2}}{{\left|\frac{1-\Gamma_{\mathrm{LMS}}\Gamma_{311}}{1-\Gamma_{\mathrm{LMS}}\Gamma_{310}}\right|}^{2}\frac{\left(1-{\left|\Gamma_{310}\right|}^{2}\right)}{\left(1-{\left|\Gamma_{311}\right|}^{2}\right)}},$$ (16)

where *Γ*_311_ is the reflection coefficient of mount three at the high level, *Γ*_310_ is the reflection coefficient of mount three at the low level, and *Γ*_LMS_ is the reflection coefficient of the system looking into port three.

The measured value of *Γ*_310_ was actually taken 10 dB below *Γ*_310_ instead of the 6 dB step, so the uncertainty should be conservative. Using nominal values for the *P*’s and the measured reflection coefficients, the calculated uncertainty is ±0.014% or ± 62 μB.

Impedance changes in the diode detector with respect to power level are assumed to have a negligible effect on the overall uncertainty.

#### 6.2.6 Uncertainty Due to RF Leakage

It is difficult to assign a quantitative uncertainty due to rf leakage. In order to reduce leakage, all coaxial cables in the system were replaced with semirigid lines and, wherever possible, SMA connectors were used. For now, the uncertainty due to rf leakage is assumed to be ±10 μB or ±0.0023%.

#### 6.2.7 Uncertainty Due to Spurious Signals and Harmonics

After the signal is amplified and filtered, any harmonics are at least −92 dBc, while spurious signals are no greater than − 84 dBc. This will result in a measurement error that will affect the uncertainty of $C_{\mathrm{HL}}^{2}$.

From Eq, (3), the calculated dc power is

$$P_{\mathrm{dc}}=20\left(V_{\text{off}}^{2}-V_{\text{on}}^{2}\right),$$ (17)

where *P*_dc_ is in mW. Assuming *V*_off_ = 1 V, then *V*_on_ = 0.63246 V for a calculated power of 12 mW and *V*_on_ = 0.92195 V for a calculated power of 3 mW.

A harmonic at −92 dBc results in an uncertainty of ±0.0025% for a voltage measurement. This uncertainty translates to power uncertainties of (12 ±0.0004) mW and (3 ±0.0008) mW. Using the nominal powers to calculate $C_{\mathrm{HL}}^{2}$, an uncertainty of ±0.023% or ±100 μB is obtained.

A spurious signal at −112 dBc results in an uncertainty of ±0.00025% for a voltage measurement. This uncertainty translates to power uncertainties of (12 ±0.00004) mW and (3 + 0.000085) mW. Using the nominal powers to calculate $C_{\mathrm{HL}}^{2}$, an uncertainty of ±0.0025% or ±11 μB is obtained.

#### 6.2.8. Overall Type B Uncertainty

For each Type B component, an estimated range, ±*a_j_*, is given, assuming that the quantity in question has a 100% probability of lying within that interval. The quantity is treated as if it is equally probable for its value to lie anywhere within the interval. Therefore, it is modeled by a rectangular probability distribution. The best estimate of the standard deviation, *u_j_* is

$$u_{j}=\frac{a_{j}}{\sqrt{3}}.$$ (18)

Table 2 shows all of the Type B components along with their corresponding uncertainties and standard deviations. The overall standard deviation of the Type B components, calculated using the root-sum-of-squares method (RSS), is ±0.069% or ±299 μB.

### 6.3 Combined Standard Uncertainty

The combined standard uncertainty, *u*_r_, is taken to represent the estimated standard deviation of the result. It is obtained by combining the individual standard deviations, *u*_i_, whether arising from a Type A or a Type B evaluation [4]. The technique used to combine the standard deviations is the RSS method.

The total uncertainty reported is the expanded uncertainty, *U*, which is obtained by multiplying *u*_c_ by a coverage factor, *k.* To be consistent with current international practice, the value of *k* used at NIST for calculating *U* is *k* = 2. The total expanded uncertainty, *U*, of the mount’s nonlinearity is calculated to be ±0.138% or ±598 μB.

## 7. Conclusion

The first step toward offering a calibration service at NIST to support rf attenuators and voltage doublers that operate at 30 MHz has been completed. The reference standard, a tuned single-element thermistor mount, has been calibrated using an LMS, designed and constructed at NIST. The next step is to modify the LMS so that a device under test may be inserted into the system and calibrated against the reference standard.

## Figures and Tables

**Fig. 5:**
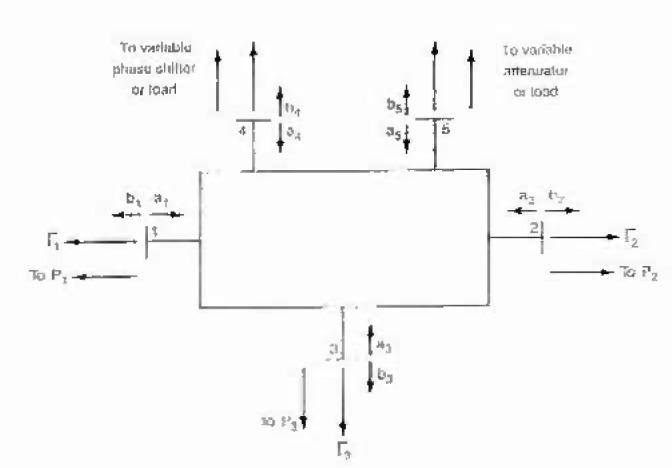
LMS port nomenclature.

**Table 1:** Sample calibration results of the 30 MHz Linear Measurement System’s standard mount

	Switches 1&2	Switch 1	Switch 2
P(1)	11.5194	11.5089	0.0000
P(2)	11.4170	0.0001	11.3991
P(3)	11.0907	2.7644	2.7756
Leg 1: 6.0335 dB			
Leg 2: 6.0160 dB			
$C_{\mathrm{HL}}^{2}:1.00029$			

**Table 2:** Type B components of the 30 MHz Linear Measurement System with corresponding uncertainty ranges and standard deviations

Component	Range (%)	Standard deviation (%)
dc voltage measurements	±0.028	0.016
Imperfect isolation	±0.112	0.065
*P*_301_≠*P*_310_	±0.0005	0.0003
Impedance changes in P_3_	±0.014	0.008
rf leakage	±0.0023	0.0013
Spurious signals	±0.0025	0.0014
Harmonics	±0.023	0.013
Combined Type B Standard Uncertainty (RSS)		± 0.069 (±299 μB)

## 8. Appendix A. Scattering Coefficient Analysis of the LMS

The 30 MHz Linear Measurement System can be considered a five port network, as shown in Fig. 5. The analysis assumes that the LMS is an ideal system—that is, the system is linear, and when *b*_1_ and *b*_2_ are assumed to be 0, they truly are.

The pertinent scattering coefficients are

$$\begin{array}{l} b_{1}=S_{11}a_{1}+S_{12}a_{2}+S_{13}a_{3}+S_{14}a_{4}+S_{15}a_{5}\\ b_{2}=S_{21}a_{1}+S_{22}a_{2}+S_{23}a_{3}+S_{24}a_{4}+S_{25}a_{5}\\ b_{3}=S_{31}a_{1}+S_{32}a_{2}+S_{33}a_{3}+S_{34}a_{4}+S_{35}a_{5}\\ b_{4}=S_{41}a_{1}+S_{42}a_{2}+S_{43}a_{3}+S_{44}a_{4}+S_{45}a_{5}\\ b_{5}=S_{51}a_{1}+S_{52}a_{2}+S_{53}a_{3}+S_{54}a_{4}+S_{55}a_{5}. \end{array}$$ (19)

If *a*_1_ *= Γ*_1_*b*_1_ and *a*_2_ = *Γ*_2_*b*_2_, then Eq. (19) may be written as

$$\begin{array}{l} b_{1}\left(1-\Gamma_{1}S_{11}\right)=S_{12}\Gamma_{2}b_{2}+S_{13}a_{3}+S_{14}a_{4}+S_{15}a_{5}\\ b_{2}\left(1-\Gamma_{2}S_{22}\right)=S_{21}\Gamma_{1}b_{1}+S_{23}a_{3}+S_{24}a_{4}+S_{25}a_{5}\\ b_{3}=S_{31}\Gamma_{1}b_{1}+S_{32}\Gamma_{2}b_{2}+S_{33}a_{3}+S_{34}a_{4}+S_{35}a_{5}\\ b_{4}=S_{41}\Gamma_{1}b_{1}+S_{42}\Gamma_{2}b_{2}+S_{43}a_{3}+S_{44}a_{4}+S_{45}a_{5}\\ b_{5}=S_{51}\Gamma_{1}b_{1}+S_{52}\Gamma_{2}b_{2}+S_{53}a_{3}+S_{54}a_{4}+S_{55}a_{5} \end{array}$$ (20)

This gives five equations and eight unknown variables–*a*_3_, *a*_4_, *a*_5_, *b*_1_, *b*_2_, *b*_3_, *b*_4_, and *b*_5_, Four variables can be eliminated from Eq. (20), say *a*_4_, *b*_4_, *a*_5_, and *b*_5_. Eq. (20) becomes

$$b_{1}=P_{13}^{\prime }a_{3}+Q_{12}^{\prime }b_{2}+Q_{13}^{\prime }b_{3},$$ (21)

where the primed quantities are functions of the scattering coefficients and *Γ*_1_i and *Γ*_2_.

It is assumed that *Γ*_3_ is constant with respect to varying power levels, If now *a*_3_ = *Γ*_3_*b*_3_,

b1=(P13′Γ3+Q13′)b3+Q12′b2≡Q13b3+Q12b2 (22)

or

$$b_{3}=\frac{b_{1}-Q_{12}b_{2}}{Q_{13}},$$ (23)

where two complex constants, *Q*_12_ and *Q*_13_, describe the relationship among *b*_1_, *b*_2_, and *b*_3._

